# *Codonopsis lanceolata* attenuates allergic lung inflammation by inhibiting Th2 cell activation and augmenting mitochondrial ROS dismutase (SOD2) expression

**DOI:** 10.1038/s41598-019-38782-6

**Published:** 2019-02-19

**Authors:** Yun-Soo Seo, Hyo Seon Kim, A. Yeong Lee, Jin Mi Chun, Sung Bae Kim, Byeong Cheol Moon, Bo-In Kwon

**Affiliations:** 1 Herbal Medicine Research Division, Korea Institute of Oriental Medicine, 1672 Yuseong-daero, Yuseong-gu, Daejeon 34054 Republic of Korea; 2 Department of Pathology, College of Korean Medicine, Sangji University, Wonju-si, Gangwon-do 26339 Republic of Korea

## Abstract

Allergic asthma is a chronic inflammatory disease induced by the inhalation of allergens, which trigger the activation of T helper type 2 (Th2) cells that release Th2 cytokines. Recently, herbal medicines are being considered a major source of novel agents to treat various diseases. In the present study, we evaluated the anti-asthmatic effects of a *Codonopsis lanceolata* extract (CLE) and the mechanisms involved in its anti-inflammatory effects. Treatment with CLE reduced infiltration of inflammatory cells, especially eosinophils, and the production of mucus in lung tissues. Levels of Th2 cytokines, such as IL-4, IL-5, and IL-13, and chemokines were also decreased following treatment with CLE. Moreover, Th2 cell proportion *in vivo* and differentiation *in vitro* were reduced as evidenced by the decreased expression of GATA3^+^. Furthermore, the expression of superoxide dismutase (SOD)2, a mitochondrial ROS (mROS) scavenger, was increased, which was related to Th2 cell regulation. Interestingly, treatment with CLE increased the number of macrophages in the lungs and enhanced the immune-suppressive property of macrophages. Our findings indicate that CLE has potential as a novel therapeutic agent to inhibit Th2 cell differentiation by regulating mROS scavenging.

## Introduction

Allergic asthma is a chronic pulmonary disease caused by an inappropriate immune response to aeroallergens in susceptible individuals. Allergic asthma is characterised by several clinical symptoms, including airway hyper-responsiveness, mucus hypersecretion, and inflammatory cell infiltration, induced by the inhalation of allergens such as pollen, house dust, inhalants, and air pollutants^1^. The allergens processed by antigen-presenting cells trigger the activation of T helper type 2 (Th2) cells that release Th2 cytokines, which induce inflammatory cell infiltration into the airways^2,3^. Excess recruitment of inflammatory cells into the airway contributes to airway hyper-responsiveness involved in the maintenance and development of allergic asthma by release of a variety of inflammatory mediators^4,5^. Recently, it has become obvious that Th2-mediated cytokines orchestrate the pathogenesis of allergic lung inflammation. During an allergic reaction, Th2 cells migrate to the lungs and secrete interleukin (IL)-4, IL-5, and IL-13. IL-4 and IL-13 induce mucus over-production, bronchoconstriction, and isotype switching of B cells leading to IgE production^6,7^. IL-5 is a key mediator of eosinophil activation, recruitment, and survival^8–10^.

During Th cell differentiation, various factors such as the type of antigen-presenting cells (APCs), co-stimulatory factors, and cytokines regulate the polarisation of naive Th cells into Th cell subsets^11^. However, the most effective inducer of CD4 T cell differentiation appears to be the local cytokine environment. IL-4 drives the differentiation into the Th2 phenotype^12,13^. IL-6, a cytokine produced by several cell types including APCs such as macrophages, dendritic cells, and B cells, promotes Th2 differentiation^14^. IL-6 induces the initial production of IL-4 in CD4 T cells, thereby polarising naive CD4 T cells into effector Th2 cell^15^. IL-6 is released during the NF-κB pathway, which is activated by various factors including reactive oxygen species (ROS)^16^.

Macrophages, which are the most abundant immune cells in the lungs, link the innate and adaptive immune systems during allergen-induced airway inflammation. Lung macrophages can be classified into alveolar macrophages and interstitial macrophages based on their location^17^. Macrophages are also classified based on their functional phenotypes—classically activated macrophages (M1) and alternatively activated macrophages (M2). M2 macrophages are further classified into three subtypes: M2a, M2b, and M2c^18^. M1 cells activate Th1 cells *via* TNFα production; M2a cells activate Th2 cells *via* IL-4 and IL-13 production, and M2c cells activate Treg cells *via* IL-10 and TGFβ production. Macrophages localised to the interstitial area of the lung appear to be less prone to polarisation toward either the M1 or the M2a phenotype, as these cells predominately express IL-10 and exhibit immunosuppressive properties similar to the M2c phenotype^19^.

Polarisation of macrophages depends on various environmental stimuli: deficiency in ROS production induces polarisation toward the M2 phenotype followed by a reduction in TNFα and IL-1β levels^20^; and deficiency in SOD levels induces an increase in alveolar macrophages with the M1 phenotype^21^.

ROS play as a key role in pathways involved in inflammatory disorders including tissue injury and dysfunction^22^. Oxidative stress can induce smooth muscle contraction, airway hyper-responsiveness, and increase mucus secretion^23–25^. Recently, the role of mitochondrial ROS (mROS) as a signalling intermediate was reported to be different from that of ROS generated by NADPH oxidase and uncoupled nitric oxide synthases that induce oxidative stress. mROS are involved in the activation of antigen-specific CD4+ T cells and the expression of cytokines such as IL-2 and IL-4^26^. Generation of mROS is required for the optimal activity of NFAT, NF-κB, and TCR-signalling, necessary for Th cell activation^27,28^. Furthermore, oxidative stress causes regulatory T cell apoptosis and depletion, thereby exacerbating inflammation^29^. The balance of mROS is controlled by generation at complex I, II and III and scavenging by anti-oxidants such as SOD2.

*Codonopsis lanceolata* has long been used as a traditional herbal medicine in northeast Asia, mainly in Korea, Japan, and China for the treatment of inflammatory diseases. Many previous reports have shown that *C. lanceolata* has anti-tumour, anti-obesogenic, anti-lipogenic, anti-inflammatory, and antioxidant effects^30–33^. Several studies have reported on the anti-inflammatory effects of *C. lanceolata* against lung inflammation, and most of them focused on the effects of *C. lanceolata* on alveolar macrophages^34,35^. The effects of *C. lanceolata* on the adaptive immune system have not been reported thus far. Therefore, in this study, we focused on the regulatory effects of a *C. lanceolata* extract (CLE) on Th2 cell activation and mROS generation.

## Results

### Chemical Profile of *C. lanceolata*

Ultra-performance liquid chromatography (UPLC) was performed to identify the main constituents of the CLE. Supplementary Fig. S1 shows the results of total ion chromatography (TIC) of CLE. Lancemaside A showed the highest peak at approximately 3.03 min. The molecular weight of lancemaside A according to selective ion recording (SIR) in the negative-ion mode was 1189 m/z, indicating that the component was a deprotonated molecule (Supplementary Fig. S1).

### CLE treatment alleviated pathological changes during allergic lung inflammation

**Figure 1:**
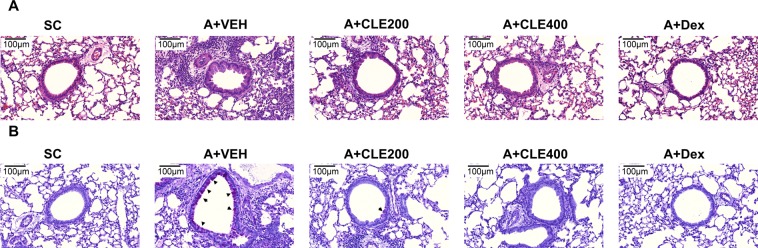
Effects of *Codonopsis lanceolata* extract (CLE) on histopathological changes in the lung. Sections of lung tissue were stained with haematoxilin and eosin (H&E) (**A**) and periodic acid–Schiff (PAS) (**B**). Arrowheads indicate mucus production. SC, saline control group; A + VEH, asthmatic lung inflammation group; A + CLE200, A + CL extract 200 mg/kg; A + CLE400, A + CL extract 400 mg/kg; A + Dex, A + dexamethasone 5 mg/kg.

Eosinophil infiltration, activation of Th2 cell and production of type 2 cytokine in asthma patients are suggested to be a contributing causative agent in the pathophysiology and lung dysfunction that are characteristic of asthma^36^. To investigate the anti-asthmatic effects of CLE, we used OVA-induced allergic inflammation model which known as a reproducible model that show various phenotypes of acute allergic asthma^37,38^. Also this model was well-established for the study about potential anti-asthmatic drug evaluation^37,38^. Briefly, after OVA sensitizations, OVA nasal administration was performed with or without CLE oral administration. To evaluate the pharmacological effects of the CLE on inflammatory cell infiltration into the peribronchiolar and perivascular lesions in lung tissue, histopathological changes were analyzed (Fig. 1). The OVA-challenged mice showed a marked increase in inflammatory cell infiltration into the peribronchiolar and perivascular lesions in the lung tissue. However, treatment with CLE led to a significant reduction in inflammatory infiltrates. To evaluate airway overproduction of mucus and goblet cell hyperplasia, lung sections were stained with periodic acid–Schiff (PAS). Goblet cell hyperplasia in the airway was clearly observed in OVA-challenged mice unlike that in control mice. CLE treatment markedly inhibited mucus hypersecretion and goblet cell hyperplasia in a dose-dependent manner in lung tissues as observed in the dexamethasone (Dex) treatment group.

### CLE treatment reduced inflammatory cell levels in the bronchoalveolar lavage fluid (BALF) in OVA-induced allergic lung inflammation

**Figure 2:**
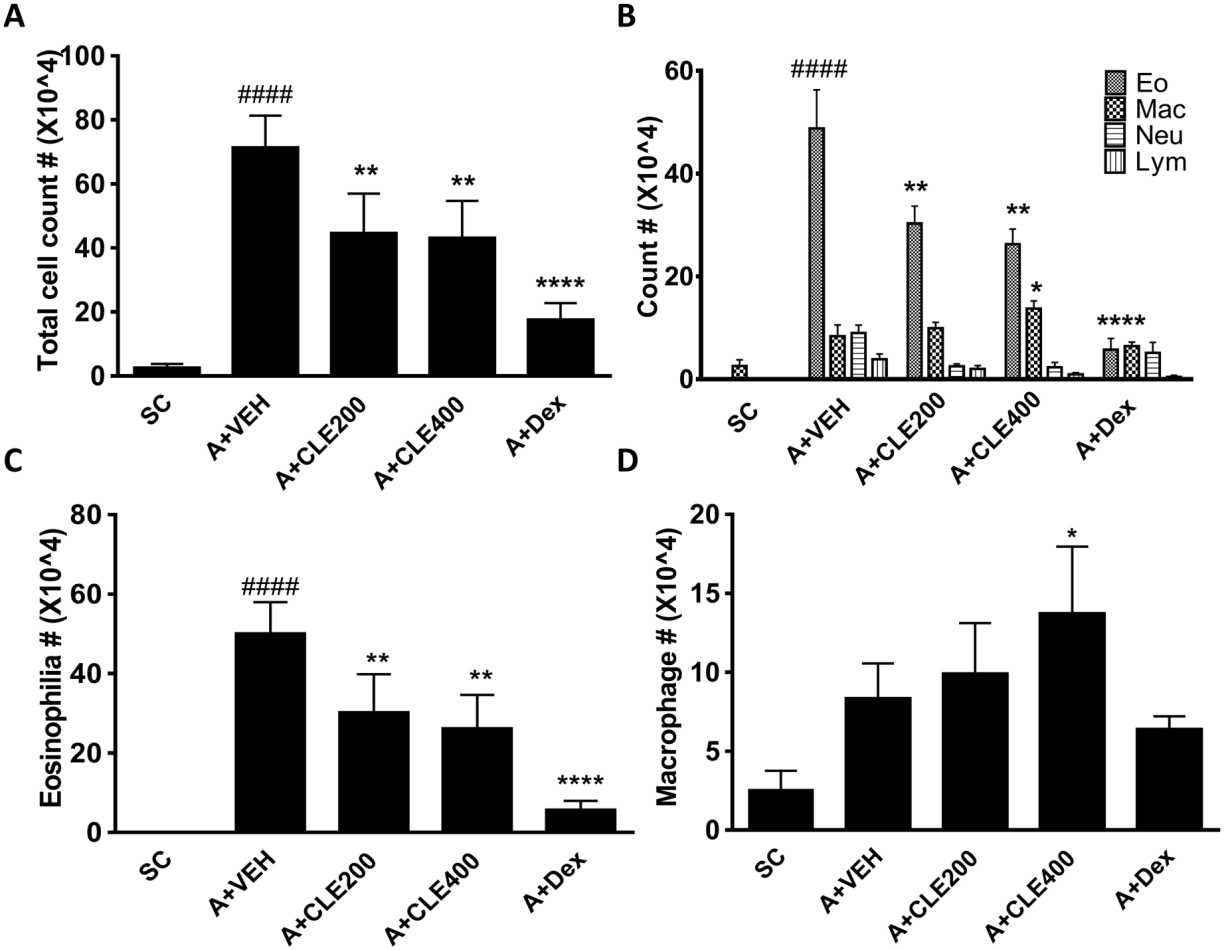
Effects of *Codonopsis lanceolata* extract (CLE) on recruitment of inflammatory cells in the BALF. BALF was collected 18 h after the last OVA challenge, and the cells were isolated by cytospin. SC, saline control group; A + VEH, asthmatic lung inflammation group; A + CLE200, A + CL extract 200 mg/kg; A + CLE400, A + CL extract 400 mg/kg; A + Dex, A + dexamethasone 5 mg/kg. ^####^*P* < 0.001 compared with the SC group. *P < 0.05, **P < 0.01, ***P < 0.005, and ****P < 0.001 compared with the allergic inflammation plus vehicle group.

In allergic airway inflammation, infiltration and activation of immune cells, especially eosinophil, were major marker of severity. To confirm that CLE inhibits inflammatory cell infiltration, especially that of eosinophils, we evaluated the number of inflammatory cells, including eosinophils, neutrophils, macrophages, and lymphocytes, in bronchoalveolar lavage fluid (BALF) (Fig. 2). In OVA-challenged mice, the number of total inflammatory cells, particularly eosinophils, was markedly higher than that in the saline-treated control mice (Fig. 2A–C). The CLE-treated mice, alike Dex-treated mice, had significantly fewer inflammatory cells, especially eosinophils, than the allergic lung inflammation mice treated with vehicle in a dose-dependent manner (Fig. 2A–C). These results indicated that CLE inhibited infiltration of inflammatory cells. Furthermore, CLE treatment, as opposed to Dex treated group, significantly increased the number of macrophages at the highest dose compared to that in control mice (Fig. 2D). As a previous study showed that alveolar macrophages have a suppressive role in allergic airway inflammation^39^, we further investigated the relation between anti-asthmatic effects of CLE and increase in the number of alveolar macrophages.

### CLE administration decreased the levels of Th2-related cytokines in BALF

**Figure 3:**
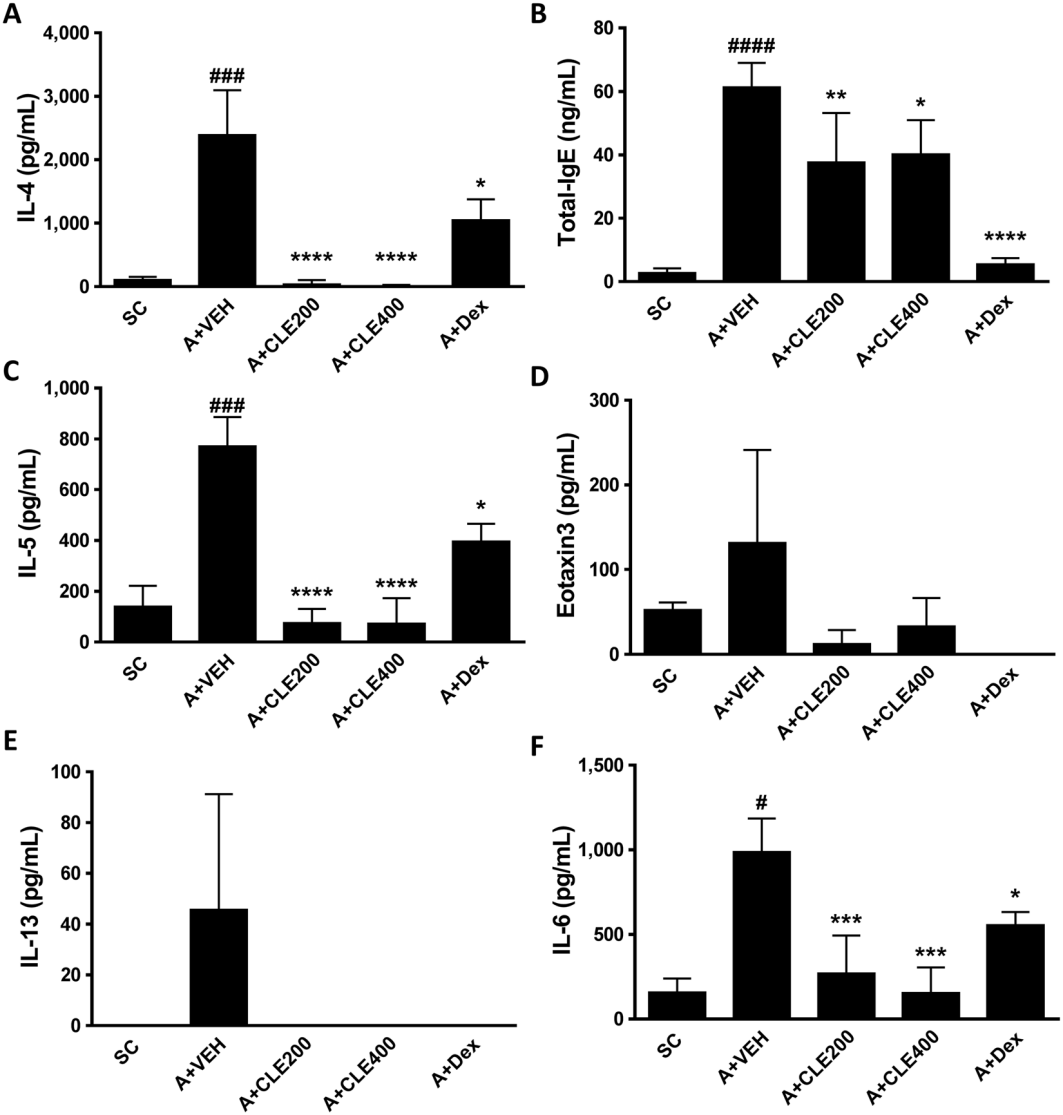
Effects of *Codonopsis lanceolata* extract (CLE) on the expression of inflammatory mediators in the BALF. BALF was collected 18 h after the last OVA challenge. SC, saline control group; A + VEH, asthmatic lung inflammation group; A + CLE200, A + CL extract 200 mg/kg; A + CLE400, A + CL extract 400 mg/kg; A + Dex, A + dexamethasone 5 mg/kg. ^#^*P* < 0.05, ^###^*P* < 0.005 and ^####^*P* < 0.001 compared with the SC group. *P < 0.05, **P < 0.01, ***P < 0.005, and ****P < 0.001 compared with the allergic inflammation plus vehicle group.

Multiple cytokines has been shown to be related to the development of allergic inflammation. Th2 cytokines, such as IL-4, 5 and 13, are known as major players in development of airway hyperresponsiveness (AHR), eosinophil infiltration, and mucus hypersecretion^40^. To identify the inhibitory effect of CLE treatment on Th2-related cytokines, we estimated cytokine levels in BALF. As shown in Fig. 3, the OVA-challenged group showed significant increases in IL-4, IL-5, IL-6, and IgE levels. IL-13 and eotaxin 3 showed only an increasing tendency in asthmatic mice. After treatment with CLE, the expression levels of Th2 cytokines, such as IL-4 and IL-5, and IL-6 were significantly lower than Dex treated group in both CLE treated groups (Fig. 3A, C and F). In addition, production of IgE, eotaxin3 and IL-13 were also reduced as observed in Dex treatment group (Fig. 3B, D and E). These results indicated that CLE attenuated the allergic inflammation mediated by activation of Th2 cells.

### CLE administration reduced Th2 cell activation in OVA-induced allergic lung inflammation and ***in vitro***

**Figure 4:**
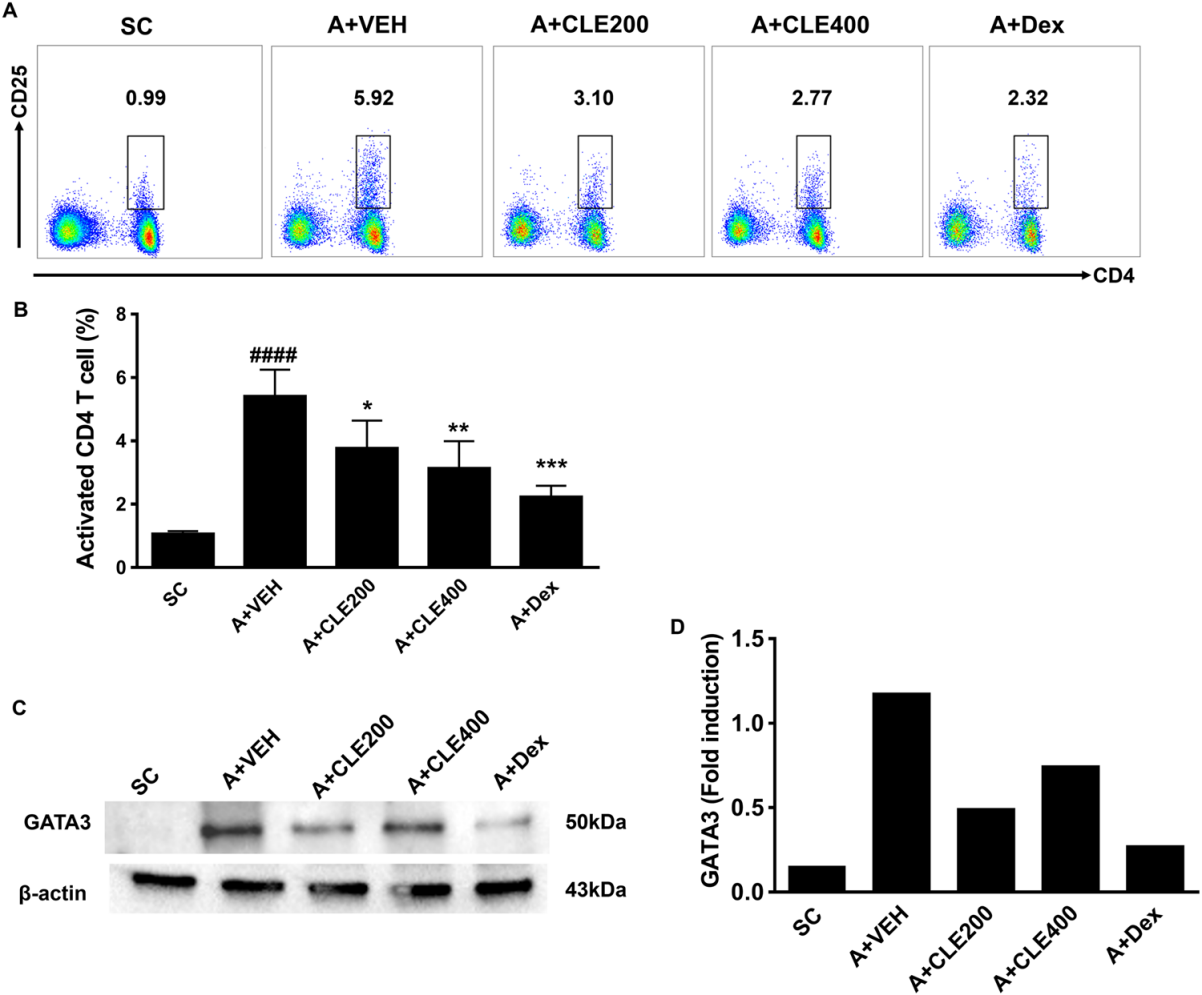
Effects of *Codonopsis lanceolata* extract (CLE) on Th cell activation in the lung tissues analysed using flow cytometry. Representative (**A**) and compiling data (**B**) are shown. The effects of CLE on the expression of GATA3 in the lung tissue using western blot analysis (**C**,**D**). Full-length blots are presented in Supplementary Fig. S4. SC, saline control group; A + VEH, asthmatic lung inflammation group; A + CLE200, A + CL extract 200 mg/kg; A + CLE400, A + CL extract 400 mg/kg; A + Dex, A + dexamethasone 5 mg/kg. ^####^*P* < 0.001 compared with the SC group. *P < 0.05, **P < 0.01, and ***P < 0.005 compared with the allergic inflammation plus vehicle group.

**Figure 5:**
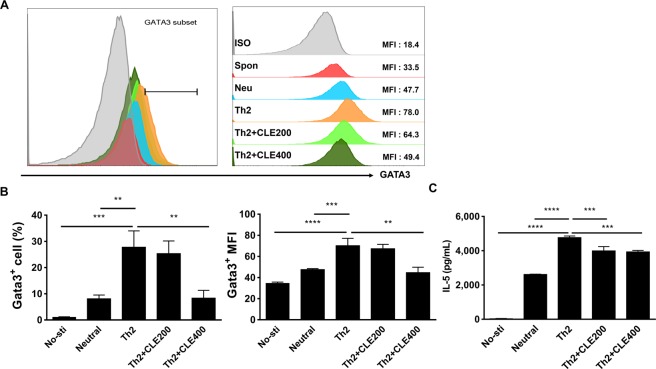
Effects of *Codonopsis lanceolata* extract (CLE) on Th2 cell activation *in vitro*. GATA3^+^ T cell population and mean fluorescence intensity (MFI) were analysed. Representative (**A**) and compiling data (**B**) are shown. IL-5 were measured by using ELISA (**C**). No-sti, without stimulation; Neutral, stimulated with CD3 and CD 28; Th2, stimulated with neu + IL2, IL-4, and anti-IFNγ; Th2 + CLE200, Th2 + CL extract 200 μg/ml; Th2 + CLE400, Th2 + CL extract 400 μg/ml. **P < 0.01, ***P < 0.005, and ****P < 0.001 compared with the Th2 polarisation condition.

We found that CLE treatment ameliorated allergic lung inflammation by inhibiting mucus production and eosinophil infiltration *via* regulation of Th2-related cytokines and allergic mediators. Therefore, we next tried to verify whether CLE regulates the activation of Th2 cells. As shown in Fig. 4, the proportion of activated T cells (CD4^+^, CD25^+^) was increased in the lungs of the asthma group compared to that of the control group. After treatment with CLE, T cell activation decreased, as observed in the Dex treatment group, in a dose-dependent manner. As shown in Fig. 4C, the increased expression of GATA3 (thus defined as th2 cell) was reduced in the asthma group following CLE treatment group. These results indicate that CLE treatment, alike in Dex treatment, improved asthmatic conditions by regulating Th2 cell activation. To confirm the inhibitory effects of CLE on Th2 cell activation *in vitro*, CD4+ T cells were isolated and polarised with or without CLE. Purified CD4+ T cells (Supplementary Fig. 3A) were cultured under neutral and Th2 polarisation conditions. Following CLE treatment, unlike that under neutral conditions, IFN-γ production was found to be low under Th2 polarisation conditions (Supplementary Fig. 3B). Treatment with CLE significantly reduced the expression of GATA3 during Th2 differentiation (Fig. 5A and B) in highest concentration. As expected, IL-5 secretion was also decreased after treatment with CLE in both concentration. These results indicate that CLE blocked Th2 cell differentiation and activation, which is considered one of the main mechanisms underlying its anti-asthmatic effects.

### CLE induced the expression of SOD2 and activated Treg cells in OVA-induced allergic lung inflammation

**Figure 6:**
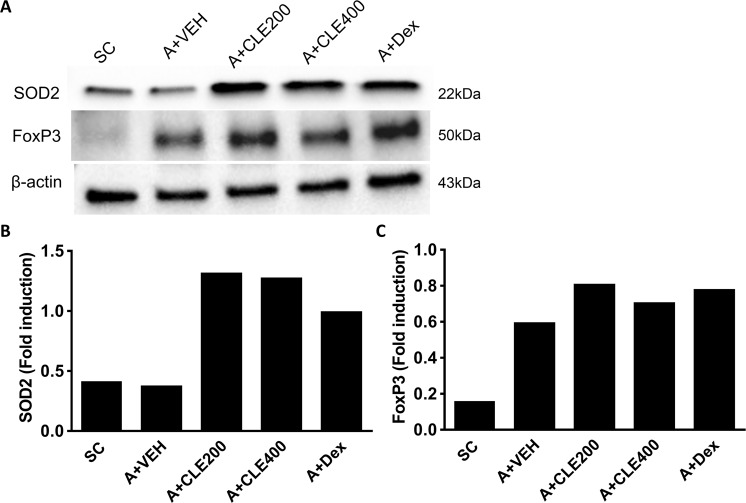
The effects of *Codonopsis lanceolata* extract (CLE) on the expression of SOD2 and FoxP3 in the lung tissues analysed using western blot. The bands were evaluated and normalized using β-actin (**B**). Full-length blots are presented in Supplementary Fig. S5. SC, saline control group; A + VEH, asthmatic lung inflammation group; A + CLE200, A + CL extract 200 mg/kg; A + CLE400, A + CL extract 400 mg/kg; A + Dex, A + dexamethasone 5 mg/kg.

To investigate the underlying mechanism of CLE-induced reduction in Th cell activation, we measured the expression levels of SOD2 in the lung of asthmatic mice (Fig. 6). There was no difference between SC group and asthma group but expression levels were increased in Dex treated group. After treatment with CLE, the expression of SOD2 increased in both concentration as observed in the Dex treatment group. Because, reduction of Treg number and proportion by mROS mediated apoptosis is recovered after scavenging of mROS^41^, we expected that treatment with CLE increased Treg population. CLE treatment also induced the expression of FoxP3, a key transcription factor involved in Treg cell function in both concentration as observed in the Dex treatment group. These results indicate that CLE induced SOD2 expression and activated Treg cells.

### CLE induced IL-10 production in BALF and *in vitro* macrophages culture system

**Figure 7:**
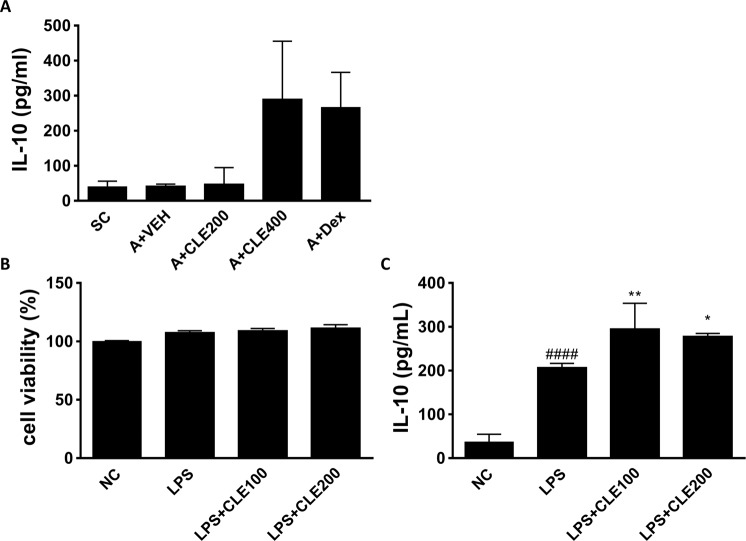
Effects of *Codonopsis lanceolata* extract (CLE) on production of IL-10 in the BALF (**A**), cell viability (**B**) and production of IL-10 (**C**) in murine macrophages. SC, saline control group; A + VEH, asthmatic lung inflammation group; A + CLE200, A + CL extract 200 mg/kg; A + CLE400, A + CL extract 400 mg/kg; A + Dex, A + dexamethasone 5 mg/kg. NC, without stimulation; LPS, stimulated with LPS (1 μg/mL); LPS + CLE200, LPS + CL extract 200 μg/mL; LPS + CLE400, LPS + CL extract 400 μg/mL. ^####^*P* < 0.001 compared with the NC group. *P < 0.05, and **P < 0.01 compared with the LPS group.

We found that the number of macrophages significantly increased compared to that of eosinophils, neutrophils, and lymphocytes in BALF. To evaluate the relation between increased number of macrophages and attenuated asthmatic phenotypes, we examined the effect of CLE on IL-10 production which known as major effector of alveolar macrophage. Expression levels of IL-10 after CLE treatment were increased in BALF in the highest concentration (Fig. 7A) as observed in the Dex treatment group and this result is consistent with increased number of alveolar macrophages in BALF as shown in Fig. 2D. Additionally, to investigate whether CLE directly modulate macrophage, we examined the effects of CLE on activated macrophages using a murine macrophage cell line. Firstly, we observed the effects of CLE on cell viability and could not detect cytotoxicity of CLE (Fig. 7B). Secondly, after stimulation with LPS, IL-10 production in RAW 264.7 cells was significantly increased compared to that in the normal control group. Treatment of activated macrophages with CLE significantly strengthened the production of IL-10 in both concentration than vehicle treated group (Fig. 7C). These results indicate that treatment with CLE enhanced the immune-suppressive properties of murine macrophages.

## Discussion

The pathophysiology of asthma is extremely complicated. Immune responses including interactions between Th2 cells and innate immune cells such as eosinophils and macrophages play an important role. The pathogenesis of OVA-induced asthma is characterised by allergen-activated secretion of Th2 cell cytokines, which induce IgE production, influx of inflammatory cells, such as eosinophils, into the lungs, mucus hyper-production, and airway hyper-responsiveness (AHR)^42^.

*C. lanceolata* has been used as food and as a traditional medicine in east Asian countries. In particular, the dried roots of *C. lanceolata* have been used for the treatment of inflammatory diseases of the respiratory system such as asthma, tonsillitis, and pharyngitis.

In this study, we showed that the administration of CLE attenuated allergic inflammation including secretion of cytokines and chemokines, mucus production, and infiltration of immune cells. Furthermore, we showed that these effects were associated with the polarisation of alveolar macrophages to the immune-suppressive (M2c) phenotype and decreased levels of mROS *via* regulation of SOD2 expression, which is involved in Th2 cell differentiation and activation.

Th2 cell differentiation and activation play critical roles in the pathogenesis of allergic airway inflammation^3^. Cytokines such as IL-4, IL-5, and IL-13 have a close relation with the phenotype of allergic asthma. Secretion of Th2 cytokines induces recruitment of inflammatory cells, production of mucus, and AHR, leading to the development of asthma^5^. After treatment with CLE, the levels of cytokines in BALF were reduced significantly. Decreased IL-4 and IL-5 secretions directly downregulated the production of IgE and eotaxin 3 expression, which attract eosinophils, one of the major markers of antigen-specific allergic inflammation. CLE administration also reduced mucus production and IL-6, which induces Th2 differentiation and inhibits Th1 differentiation.

To investigate the mechanism involved in CLE-induced downregulation of Th2 cell activation, we focused on mROS production, which is associated with the TCR signalling pathway and Th cell activation^28^. CLE treatment significantly increased the expression of SOD2, which is known to inhibit ROS in the mitochondria. Reduced mROS induced downregulation of Th2 cell differentiation and secretion of Th2 cytokines^43^. Furthermore, mROS regulation was related to the expression of FoxP3, which is a major transcription factor of Treg cells. Attenuation of allergic asthma by CLE involved decreased Th2 cell and increased Treg cell activation *via* regulation of mROS.

Interestingly, CLE significantly increased the number of infiltrated macrophages in the BALF. M2c macrophages activate Treg cells *via* IL-10 and TGFβ production mediated by CCL24 and MRC1, which induce the development of allergic tolerance and decrease inflammation^18^. IL-10, which is a major marker of the M2c phenotype, was significantly induced by treatment with CLE. This suggests that the increased macrophage proportion in BALF after CLE treatment attenuate the allergic asthma by induction of immune tolerance and activation of Treg cells.

The roots of *C. lanceolata* are known to contain lancemaside A as a major component^44–46^. This was confirmed in the present study by using UPLC. It has been reported that this triterpenoid saponin reduces inflammation mediated by monocytes and macrophages^44,47^. At present, it is unclear whether other compounds present in CLE also play an effective role in the regulation of macrophages or whether lancemaside A is the only active compound of CLE that acts against asthma. Additional studies are warranted in this regard with special focus on the effects of lancemaside A on mROS scavenging and helper T cell differentiation and activation.

Asthma has long been considered as a Th2 disease of the airway. However, recently, asthma has been further classified as eosinophilic asthma and neutrophilic asthma^48^. Eosinophilic asthma involves the combined action of Th2 cells, type 2 innate lymphoid cells (ILC2 cells) and basophils. ILC2 is considered as a mediator of type 2 inflammation *via* IL-13 production and efficient induction of Th2 cells^49^. Neutrophilic asthma is controlled by Th17 cells, which induce tissue injury and remodelling. CLE treatment attenuates type 2 inflammation and infiltration of eosinophils and neutrophils. Future comprehensive studies on the inhibitory effects of CLE against ILC2 or neutrophilic asthma would provide useful information.

Our study has a few limitations. We studied only the effects of CLE on mROS indirectly by evaluating SOD2 expression. The exact mechanisms underlying the association of TCR signalling, mROS, and T cell differentiation with allergic asthma were not evaluated. Moreover, the *in vitro* method used to evaluate macrophage functional phenotype switching requires further validation. In detail, experiments on Akt/mTOR pathway would be helpful to understand exact mechanism of CLE on T cell differentiation mediated by mROS production which known as a downstream of TCR signalling^28^. Experiments about macrophage differentiation using bone marrow under various cytokine condition would be helpful to explain the underlying mechanism of CLE on macrophage including polarization during differentiation and phenotype switching from M1 to M2c after differentiation. Nevertheless, despite these limitations, our study showed that CLE attenuates allergic asthma by inhibiting Th2 cell activation mediated by mROS. CLE also enhanced the immune-suppressive properties of macrophages. These results provide pharmacological evidence for the therapeutic effects and its mechanism of *C. lanceolata*, which has been used as a traditional medicine for decades.

## Materials and Methods

### Preparation of an ethanolic extract from *C. lanceolata* root

Commercial Codonopsis Lanceolatae Radix was purchased from Kwangmyongdang Co. (Ulsan, Korea) and was authenticated by microscopic and macroscopic evaluation of its characteristics (Dr. Goya Choi). The taxonomic origin of these samples was also identified by using a multiplexed sequence characterised amplified region (SCAR) marker at the species level as described previously^50^. A voucher specimen was deposited at the Korean Herbarium of Standard Herbal Resources (IH herbarium code KIOM, specimen no. 2–15–0710) at KIOM. Dried roots of *C. lanceolata* (500 g) were prepared by reflux after extraction in 70% ethanol (v/v) twice for 3 h and then concentrated under reduced pressure (yield: 33.6%).

### Ultra-performance liquid chromatography analysis

Chromatographic analysis of *C. lanceolata* was performed on an UPLC–photodiode array–quadrupole detector (QDa) instrument. The UPLC system (Acquity™ UPLC system; Waters Corporation, Milford, MA, USA) comprised a binary solvent pump, sample manager, column oven, online degasser, PDA detector, and QDa detector. Separation was performed as described previously^51^ using a YMC–Pack Pro C_18_ RS column (2.0 id × 100 mm, 3 μm; YMC, Kyoto, Japan). The mobile phase was 0.1% formic acid containing distilled water (A) and acetonitrile (B). The conditions included an isocratic mobile phase comprising 33% acetonitrile for 10 min at a flow rate of 0.2 mL/min and an injection volume of 2 μL. Nitrogen was used as the carrier gas for the QDa detector under the following conditions: capillary voltage, 0.8 V; probe temperature, 600 °C; sampling frequency, 8 Hz; cone voltage, 15 V; source temperature, 120 °C; and turbo temperature, 45 °C. Monitoring was carried out in the negative mode at *m/z* 900–1250 using the TIC, and the (SIR) of lancemaside A was detected at *m/z* 1189 in negative mode. The UPLC data were processed with Empower™ 3 software (Waters Corporation).

### Asthmatic model establishment and treatment

Seven-week-old female Balb/C mice were purchased from Daehan Biolink Co., Ltd. (Chungcheongbuk-do, Korea) and housed under specific pathogen-free conditions supplied with standard rodent feed and tap water ad libitum. The mice were divided into five groups (n = 7 per group) and subsequently inoculated with OVA to induce lung inflammation: normal control group, OVA group (vehicle treatment), OVA + CLE (200 and 400 mg/kg) treatment group, and OVA + dexamethasone (Dex) (5 mg/kg) group. OVA (Chicken egg ovalbumin; Sigma-Aldrich, St. Louis, MO) was precipitated in aluminium sulphate (Alum; InvivoGen, San Diego, CA) as described previously^52^. On days 0 and 7, the mice were sensitised by an intra-peritoneal injection with 200 μL of OVA/Alum (50 μg). Beginning on day 15, the mice were challenged *via* intranasal inhalation with 25 μg of OVA in 50 μL phosphate-buffered saline (PBS) once a day for 4 consecutive days under anaesthesia using 2% isoflurane (Piramal Ciritical Care Inc., Bethlehem, PA) delivered by Vevo^TM^ Compact Anesthesia System (FUJIFILM VisualSonics, Toronto, Canada) (Supplementary Fig. S2). The four allergic lung inflammation groups were treated orally with vehicle, CLE (200 mg/kg, 400 mg/kg), or Dex for the last 7 days. The doses of current study were chosen based on USFDA and previous study with similar design^53,54^. Briefly, based on traditional dosage (60 g/10 day), we calculated the dose for mice according to USFDA guideline and yield (33.6%), 2 g/60 kg (33.2 mg/kg) for human is equivalent to that of dose of 400 mg/kg for mouse^55,56^. Dexamethasone, a drug belong to corticosteroid, has been used for positive control as standard for inhibiting allergic asthma including immune cell infiltration including eosinophil, AHR, IgE secretion and Th2 cytokine production^57,58^. All animal care and experimental procedures were performed with the approval, of the Animal Care committee of the Korean Institute of Oriental Medicine (17–034) and all methods were confirmed in accordance with the relevant guidelines and regulations by KIOM.

### Histological analysis

Mice were sacrificed by injecting an overdose of sodium pentobarbital according to the IACUC guidelines. The collected lung tissue samples of mice were fixed in 10% formalin, embedded in paraffin, and cut into 5-μm-thick sections using a microtome (Leica, Nussloch, Germany). The sections were deparaffinised and stained with haematoxylin and eosin (H&E) to analyse inflammatory changes and with PAS to evaluate mucus production. The tissue slides were viewed under a microscope and photographed (Olympus, Olympus Optical Co., Tokyo, Japan).

### Collection of BALF and differential counting

BALF was collected by intubation through an endotracheal catheter (Jelco, St. Paul, MN) and lavaged with 1 mL Dulbecco’s PBS (Sciencell Research Laboratories, Carlsbad, CA, USA) for differential cell counting and measurement of cytokines. The cells in the BALF were counted, applied to a slide *via* cytospinning, and then stained with haema 3 solution (Fisher HealthCare, Pittsburgh, PA). After estimating the ratio of each leukocyte, the number of each cell type was calculated by multiplying the proportions by the total cell count. To separate the cells, BALF was first centrifuged for 5 min at 13,000 rpm, 4 °C, and then the supernatant was stored at −80 °C.

### Measurement of cytokines, IgE, and eotaxin 3 in BALF

The levels of IL-4, IL-5, IL-6, IL-10 and IL-13 in BALF were measured using ELISA as described previously^38^. Briefly, antibodies 11B11, TRFK5, and eBio13A were used to capture IL-4, IL-5, and IL-13, respectively, and biotinylated antibodies BVD6-24G2, TRFK4, and eBio1316H were used for detection. The antibodies were purchased from BD Biosciences (San Diego, CA), except for eBio13A and eBio1316H, which were purchased from eBioscience. The level of IL-10 was measured using Mouse IL-10 ELISA Set purchased from BD Biosciences. The level of eotaxin 3 was measured by using a BMS5008ELISA kit (eBioscience) according to the manufacturer’s instructions. Levels of total IgE in BALF were measured using an ELISA kit 439807 (BioLegend, San Diego, CA) according to the manufacturer’s instructions.

### Flow cytometry

Flow cytometry was performed as previously described^59^. Briefly, fluorescein isothiocyanate (FITC)-conjugated anti-CD4 (GK1.5), phycoerythrin (PE)-conjugated anti-CD25 (PC61), PE-conjugated anti-GATA3 (16E10A23), peridinin chlorophyll (Percp)-conjugated anti-CD3 (145-2C11) (all from Biolegend, except for anti-CD3, which was from BD Biosciences) were used to stain single cell suspensions. Data were collected using the Canto II LSRFortessa X-20 instrument (BD Biosciences) and analysed using FlowJo software (TreeStar, Ashland, OR, USA).

### Western blot analysis

Lung tissues collected from saline- or allergen-challenged mice were lysed with RIPA buffer (Cell signaling, Billerica, MA). The lysates (30 μg) were separated by 10% SDS-PAGE and transferred onto nitrocellulose membranes. The membranes were blocked with 5% skimmed milk and incubated at 4 °C overnight with primary antibodies against GATA3 (0.5 μg/mL, BD Biosciences), Foxp3 (1.0 μg/mL, Abcam), and SOD2 (1:1000 dilutons, Cell signaling). After washing, the membranes were incubated with peroxidase-conjugated second antibody (Santa Cruz Biotechnology). Signals were developed with ECL select (GE Healthcare, Buckinghamshire, UK), detected using ChemiDoc (Bio-RAD, Hercules, CA), and quantified based on the intensities of the band, using Image J software (National Institute of Health, Bethesda, MD).

### *In vitro* Th2 polarisation assay and intracellular cytokine staining

CD4+ T cells were isolated from splenocytes using a CD4+ T cell isolation kit (Miltenyi Biotec, Bergisch-Gladbach, Germany) according to the manufacturer’s instructions. Purified cells were stimulated with anti-CD3/28 pre-coated particles in the absence (neutral condition) or presence of IL-2, IL-4, and anti-IFNγ ab (Th2 polarisation condition) for 5 days. The cells were treated with CLE during Th2 polarisation. After cultivation, Th2 polarisation was analysed by measuring the levels of type 2 cytokines such as IL-5 and GATA3^+^ CD4 T cell proportion. For GATA3 staining, the cells were fixed and permeabilised with the Foxp3/transcription factor staining buffer as per the manufacturer’s instructions (eBioscience) and stained with antibodies. Stained cells were analysed using flow cytometry.

### Cell culture

RAW 264.7 cells were obtained from the Korea Research Institute of Bioscience and Biotechnology (Seoul, Korea) and cultured in RPMI 1640 medium supplemented with 10% foetal bovine serum and 100 U/ml of penicillin/streptomycin sulphate. The cells were cultured in a humidified incubator with 5% CO_2_ atmosphere at 37 °C. The effects of CLE against macrophages were evaluated as described previously^60^. Briefly, to stimulate the cells, the medium was replaced with fresh RPMI 1640 medium followed by the addition of LPS in the presence or absence of CLE for 24 h. The MTS assay was used to determine the viability. Levels of IL-10 in the culture media were quantified by using ELISA kits, according to the manufacturer’s instructions.

### Statistics

Data are presented as means ± standard error of means (SEM). Differences were assessed by using ANOVA, and significance was set at *P* < 0.05. All the statistical analyses were carried out by using GraphPad Prism Software version 6.0 for Windows (GraphPad Software, La Jolla, CA).

## Supplementary information

Supplementary information

## References

[1] Lee MY (2011). Anti-asthmatic effects of Angelica dahurica against ovalbumin-induced airway inflammation *via* upregulation of heme oxygenase-1. Food and chemical toxicology: an international journal published for the British Industrial Biological Research Association.

[2] Jeon CM (2014). Siegesbeckia glabrescens attenuates allergic airway inflammation in LPS-stimulated RAW 264.7 cells and OVA induced asthma murine model. Int Immunopharmacol.

[3] Leigh R (2004). Type 2 cytokines in the pathogenesis of sustained airway dysfunction and airway remodeling in mice. Am J Respir Crit Care Med.

[4] Hansbro PM (2013). Th2 cytokine antagonists: potential treatments for severe asthma. Expert Opin Investig Drugs.

[5] Bloemen K (2007). The allergic cascade: review of the most important molecules in the asthmatic lung. Immunol Lett.

[6] Umetsu DT, DeKruyff RH (1997). TH1 and TH2 CD4+ cells in human allergic diseases. J Allergy Clin Immunol.

[7] Wills-Karp M (1998). Interleukin-13: central mediator of allergic asthma. Science.

[8] Sanderson CJ (1992). Interleukin-5, eosinophils, and disease. Blood.

[9] Simon D, Braathen LR, Simon HU (2004). Eosinophils and atopic dermatitis. Allergy.

[10] Lopez AF (1988). Recombinant human interleukin 5 is a selective activator of human eosinophil function. The Journal of experimental medicine.

[11] Kalinski P, Hilkens CM, Wierenga EA, Kapsenberg ML (1999). T-cell priming by type-1 and type-2 polarized dendritic cells: the concept of a third signal. Immunol Today.

[12] Le Gros G, Ben-Sasson SZ, Seder R, Finkelman FD, Paul WE (1990). Generation of interleukin 4 (IL-4)-producing cells *in vivo* and *in vitro*: IL-2 and IL-4 are required for *in vitro* generation of IL-4-producing cells. The Journal of experimental medicine.

[13] Swain SL, Weinberg AD, English M, Huston G (1990). IL-4 directs the development of Th2-like helper effectors. Journal of immunology (Baltimore, Md.: 1950).

[14] Diehl S, Rincon M (2002). The two faces of IL-6 on Th1/Th2 differentiation. Mol Immunol.

[15] Rincon M, Anguita J, Nakamura T, Fikrig E, Flavell RA (1997). Interleukin (IL)-6 directs the differentiation of IL-4-producing CD4+ T cells. The Journal of experimental medicine.

[16] Zhao Y (2009). Regulation of COX-2 expression and IL-6 release by particulate matter in airway epithelial cells. Am J Respir Cell Mol Biol.

[17] Saradna A, Do DC, Kumar S, Fu QL, Gao P (2018). Macrophage polarization and allergic asthma. Transl Res.

[18] Jiang Z, Zhu L (2016). Update on the role of alternatively activated macrophages in asthma. J Asthma Allergy.

[19] Song X, Xie S, Lu K, Wang C (2015). Mesenchymal stem cells alleviate experimental asthma by inducing polarization of alveolar macrophages. Inflammation.

[20] Tan HY (2016). The Reactive Oxygen Species in Macrophage Polarization: Reflecting Its Dual Role in Progression and Treatment of Human Diseases. Oxid Med Cell Longev.

[21] He C, Ryan AJ, Murthy S, Carter AB (2013). Accelerated development of pulmonary fibrosis *via* Cu, Zn-superoxide dismutase-induced alternative activation of macrophages. J Biol Chem.

[22] Mittal M, Siddiqui MR, Tran K, Reddy SP, Malik AB (2014). Reactive oxygen species in inflammation and tissue injury. Antioxid Redox Signal.

[23] Nabe T (2012). Regulatory role of antigen-induced interleukin-10, produced by CD4(+) T cells, in airway neutrophilia in a murine model for asthma. Eur J Pharmacol.

[24] Zuo L, Nogueira L, Hogan MC (2011). Reactive oxygen species formation during tetanic contractions in single isolated Xenopus myofibers. J Appl Physiol (1985).

[25] Katsumata U (1990). Oxygen radicals produce airway constriction and hyperresponsiveness in anesthetized cats. Am Rev Respir Dis.

[26] Kaminski MM (2010). Mitochondrial reactive oxygen species control T cell activation by regulating IL-2 and IL-4 expression: mechanism of ciprofloxacin-mediated immunosuppression. Journal of immunology (Baltimore, Md.: 1950).

[27] Gill T, Levine AD (2013). Mitochondria-derived hydrogen peroxide selectively enhances T cell receptor-initiated signal transduction. J Biol Chem.

[28] Sena LA, Chandel NS (2012). Physiological roles of mitochondrial reactive oxygen species. Mol Cell.

[29] Ma X (2007). A high-fat diet and regulatory T cells influence susceptibility to endotoxin-induced liver injury. Hepatology.

[30] Wang L, Xu ML, Hu JH, Rasmussen SK, Wang MH (2011). Codonopsis lanceolata extract induces G0/G1 arrest and apoptosis in human colon tumor HT-29 cells–involvement of ROS generation and polyamine depletion. Food and chemical toxicology: an international journal published for the British Industrial Biological Research Association.

[31] Lee JS (2014). Codonopsis lanceolata extract prevents diet-induced obesity in C57BL/6 mice. Nutrients.

[32] Cha A (2012). Antilipogenic and anti-inflammatory activities of Codonopsis lanceolata in mice hepatic tissues after chronic ethanol feeding. J Biomed Biotechnol.

[33] Lee YJ (2013). Antioxidant activity and anti-adipogenic effects of wild herbs mainly cultivated in Korea. Molecules.

[34] Seung-Ha Lee H-JC (2017). Comparison of Anti-asthmatic Activity by Native Codonopsis lanceolata Extract. Journal of Life Science.

[35] Joh EH, Gu W, Kim DH (2012). Echinocystic acid ameliorates lung inflammation in mice and alveolar macrophages by inhibiting the binding of LPS to TLR4 in NF-kappaB and MAPK pathways. Biochem Pharmacol.

[36] Jacobsen EA, Ochkur SI, Lee NA, Lee JJ (2007). Eosinophils and asthma. Current allergy and asthma reports.

[37] Kandhare AD, Bodhankar SL, Singh V, Mohan V, Thakurdesai PA (2013). Anti-asthmatic effects of type-A procyanidine polyphenols from cinnamon bark in ovalbumin-induced airway hyperresponsiveness in laboratory animals. Biomedicine & Aging Pathology.

[38] Chun JM (2018). Peucedanum japonicum extract attenuates allergic airway inflammation by inhibiting Th2 cell activation and production of pro-inflammatory mediators. J Ethnopharmacol.

[39] Careau E (2006). Antigen sensitization modulates alveolar macrophage functions in an asthma model. Am J Physiol Lung Cell Mol Physiol.

[40] Dunican EM, Fahy JV (2015). The Role of Type 2 Inflammation in the Pathogenesis of Asthma Exacerbations. Ann Am Thorac Soc.

[41] Chang JH, Kim YJ, Han SH, Kang CY (2009). IFN-gamma-STAT1 signal regulates the differentiation of inducible Treg: potential role for ROS-mediated apoptosis. Eur J Immunol.

[42] Liu J (2013). Effects of taraxasterol on ovalbumin-induced allergic asthma in mice. J Ethnopharmacol.

[43] Kwon BI (2017). Enhanced Th2 cell differentiation and function in the absence of Nox2. Allergy.

[44] Kim EJ (2014). Lancemaside A from Codonopsis lanceolata Modulates the Inflammatory Responses Mediated by Monocytes and Macrophages. Mediators of Inflammation.

[45] Ichikawa M (2008). Rapid identification of triterpenoid saponins in the roots of Codonopsis lanceolata by liquid chromatography-mass spectrometry. J Nat Med.

[46] Joh EH, Kim DH (2010). A sensitive liquid chromatography-electrospray tandem mass spectrometric method for lancemaside A and its metabolites in plasma and a pharmacokinetic study in mice. J Chromatogr B Analyt Technol Biomed Life Sci.

[47] Joh EH, Kim DH (2010). Lancemaside A inhibits lipopolysaccharide-induced inflammation by targeting LPS/TLR4 complex. J Cell Biochem.

[48] Lambrecht BN, Hammad H (2015). The immunology of asthma. Nat Immunol.

[49] McKenzie AN (2014). Type-2 innate lymphoid cells in asthma and allergy. Ann Am Thorac Soc.

[50] Moon, B. C. *et al*. Differentiating Authentic Adenophorae Radix from Its Adulterants in Commercially-Processed Samples Using Multiplexed ITS Sequence-Based SCAR Markers. *Appl Sci-Basel* **7**, 10.3390/app7070660 (2017).

[51] Ichikawa M (2009). Simultaneous determination of seven saponins in the roots of Codonopsis lanceolata by liquid chromatography-mass spectrometry. J Nat Med.

[52] Lee SH (2003). Differential requirement for CD18 in T-helper effector homing. Nature medicine.

[53] Nair AB, Jacob S (2016). A simple practice guide for dose conversion between animals and human. J Basic Clin Pharm.

[54] Lee AR (2017). Reduced allergic lung inflammation by root extracts from two species of Peucedanum through inhibition of Th2 cell activation. J Ethnopharmacol.

[55] https://baike.baidu.com/item/山海螺.

[56] Guangxian C. Syntax of referencing in *huanayaowuzhi* (ed. Guangxian C.) (hunankejichubanshe, 2004).

[57] Reber LL (2012). A dissociated glucocorticoid receptor modulator reduces airway hyperresponsiveness and inflammation in a mouse model of asthma. Journal of immunology (Baltimore, Md.: 1950).

[58] Yu QL, Chen Z (2018). Establishment of different experimental asthma models in mice. Exp Ther Med.

[59] Kwon BI (2013). Innate type 2 immunity is associated with eosinophilic pleural effusion in primary spontaneous pneumothorax. Am J Respir Crit Care Med.

[60] Seo YS (2017). Araliasaponin II isolated from leaves of Acanthopanax henryi (Oliv.) Harms inhibits inflammation by modulating the expression of inflammatory markers in murine macrophages. Mol Med Rep.

