# Supplementary material for: Codonopsis lanceolata attenuates allergic lung inflammation by inhibiting Th2 cell activation and augmenting mitochondrial ROS dismutase (SOD2) expression
Source: Sci Rep. 2019 Feb 19;9:2312. doi: 10.1038/s41598-019-38782-6 (PMC6381190; doi:10.1038/s41598-019-38782-6)
Supplement: Supplementary file 1 — Supplementary information [file 41598_2019_38782_MOESM1_ESM.docx]

***Codonopsis lanceolata* attenuates allergic lung inflammation by inhibiting Th2 cell activation and augmenting mitochondrial ROS dismutase (SOD2) expression.**

Yun-Soo Seo^a^, Hyo Seon Kim^a^, A Yeong Lee^a^, Jin Mi Chun^a^, Sung bae Kim^a^, Byeong Cheol Moon^a^, and Bo-In Kwon^ab*^

^a^ *Herbal Medicine Research Division, Korea Institute of Oriental Medicine, 1672 Yuseong-daero, Yuseong-gu, Daejeon, 34054, Republic of Korea*

*^b^Department of Pathology, College of Korean Medicine, Sangji University, Wonju-si, Gangwon-do,* 26339*, Republic of Korea*

*Corresponding author

Correspondence and requests for reprints should be addressed to:

Bo-In Kwon, MD (Korean medicine), PhD

Department of Pathology, College of Korean medicine, Sangji University

Wonju-si, Gangwon-do, 26339, Republic of Korea

Tel.: 82-33-730-0662; Fax: 82-33-730-0654; E-mail: kbi34812@sangji.ac.kr

**
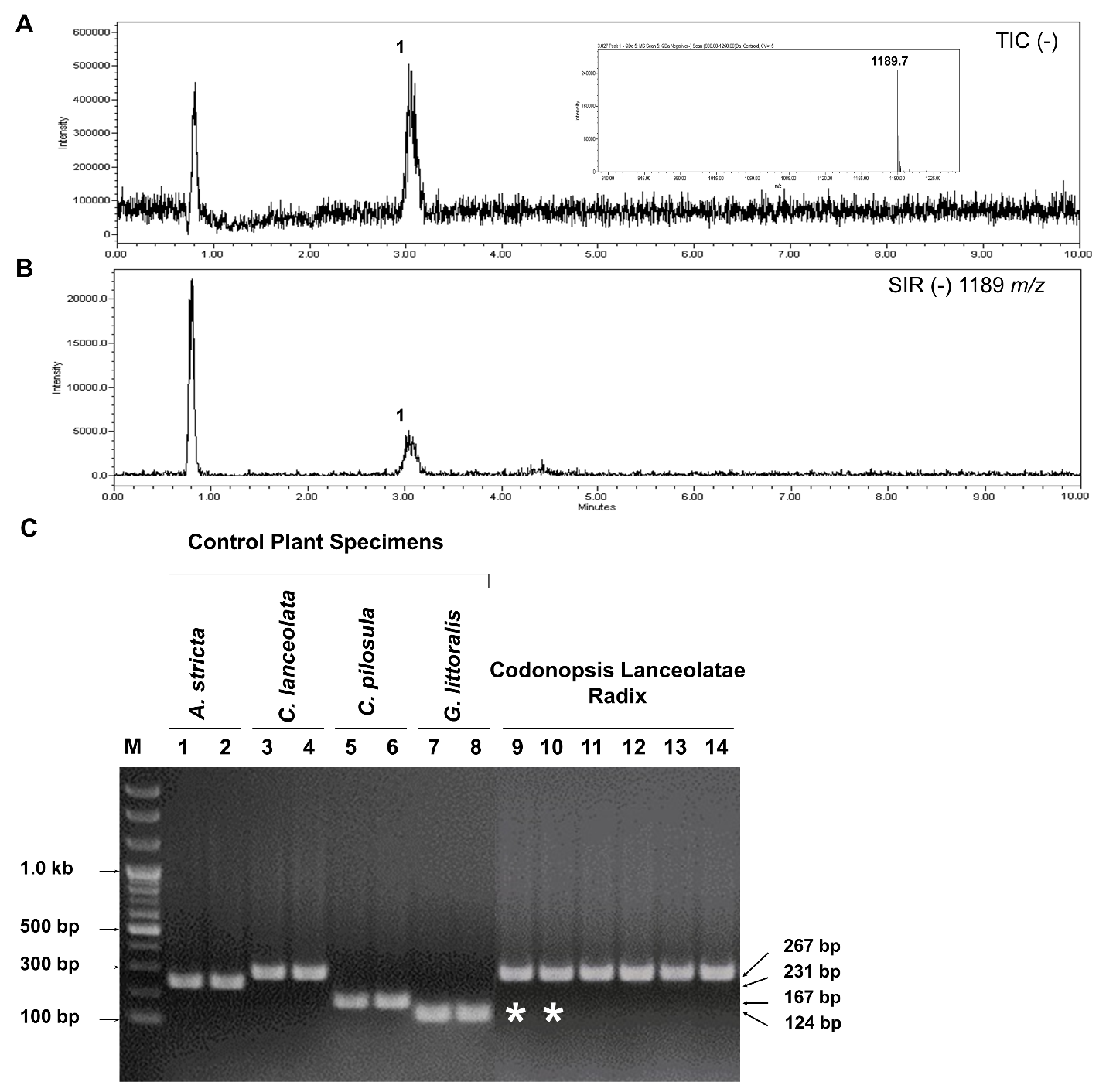
**

**Supplementary Figure S1. Authentication of the taxonomic origin of commercial medicinal materials using Ultra-performance liquid chromatography (**UPLC)–photodiode array–quadrupole detector (QDa) **chromatograms (A, B) and a multiplexed sequence characterised amplified region (SCAR) marker (C). UPLC–QDa chromatograms of the 70% ethanol extract of *Codonopsis lanceolata* roots; (A) total ion chromatography (TIC) scanning from a range of 900-1250 m/z in negative mode and mass spectrum of peak 1; (B) selected ion recording (SIR) at 1189 m/z in negative mode; peak 1, lancemaside A. (C) Lanes 1-8: control plant specimens. Lanes 9-14: commercial Codonopsis Lanceolatae Radix purchased from herbal markets. The precise sizes of the 100 bp DNA ladder and SCAR markers are indicated to the left and right side of the gel images. Asterisks (*) indicate the samples used in this study.**

**
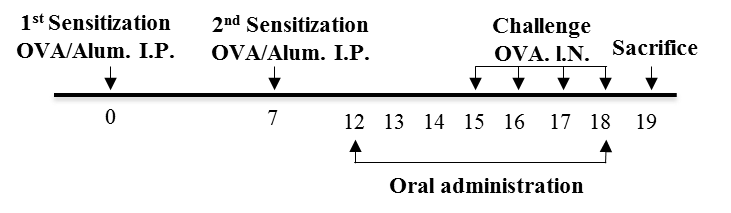
**

**Supplementary Figure S2. Asthmatic model establishment and treatment**

**
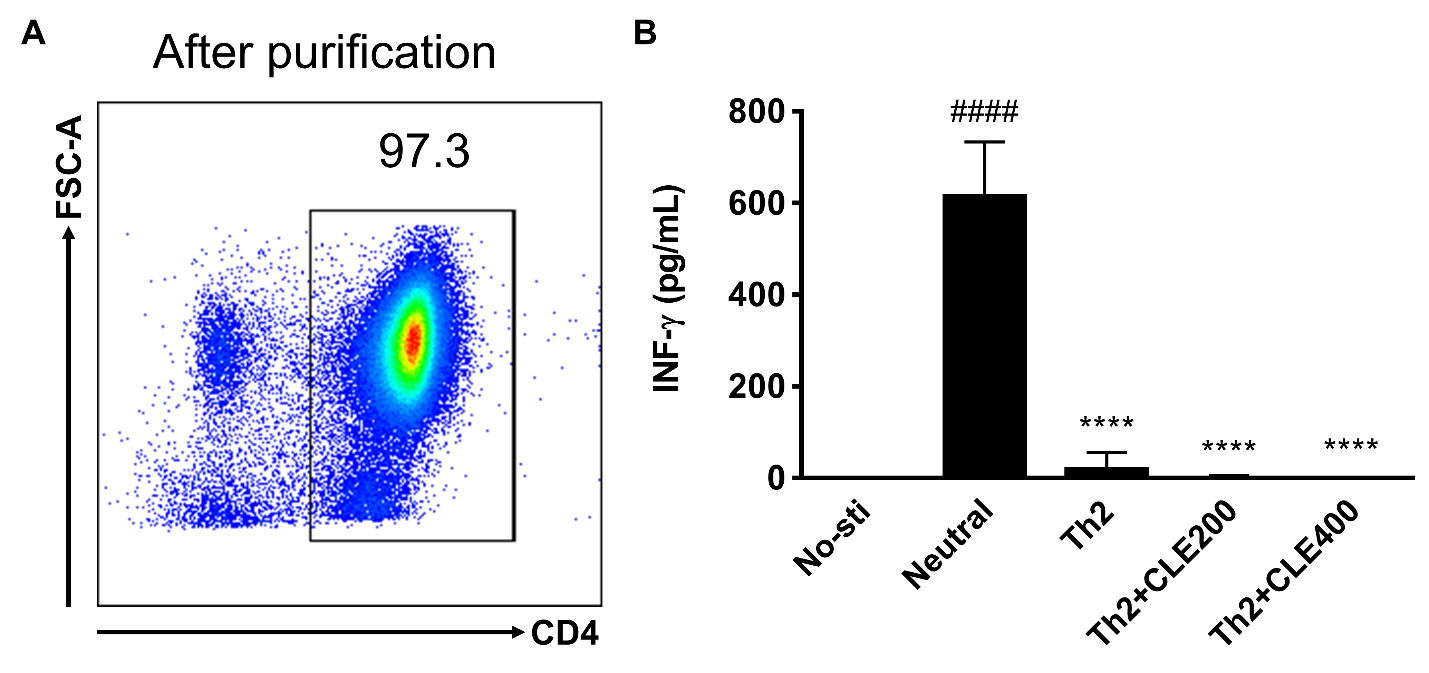
**

**Supplementary Figure S3. Purity of CD4+ T cells after purification with an CD4 T cell isolation kit (A) and production of IFNγ with or without stimuli (B). No-sti, without stimulation; Neutral, stimulated with CD3 and CD 28; Th2, stimulated with neu+IL2, IL-4, and anti-IFNγ; Th2+CLE200, Th2+ CL extract 200 μg/ml; Th2+CLE400, Th2+ CL extract 400 μg/ml. ^####^*P* < 0.001 compared with the No-sti group. *****P* < 0.001 compared with the neutral group.**

**
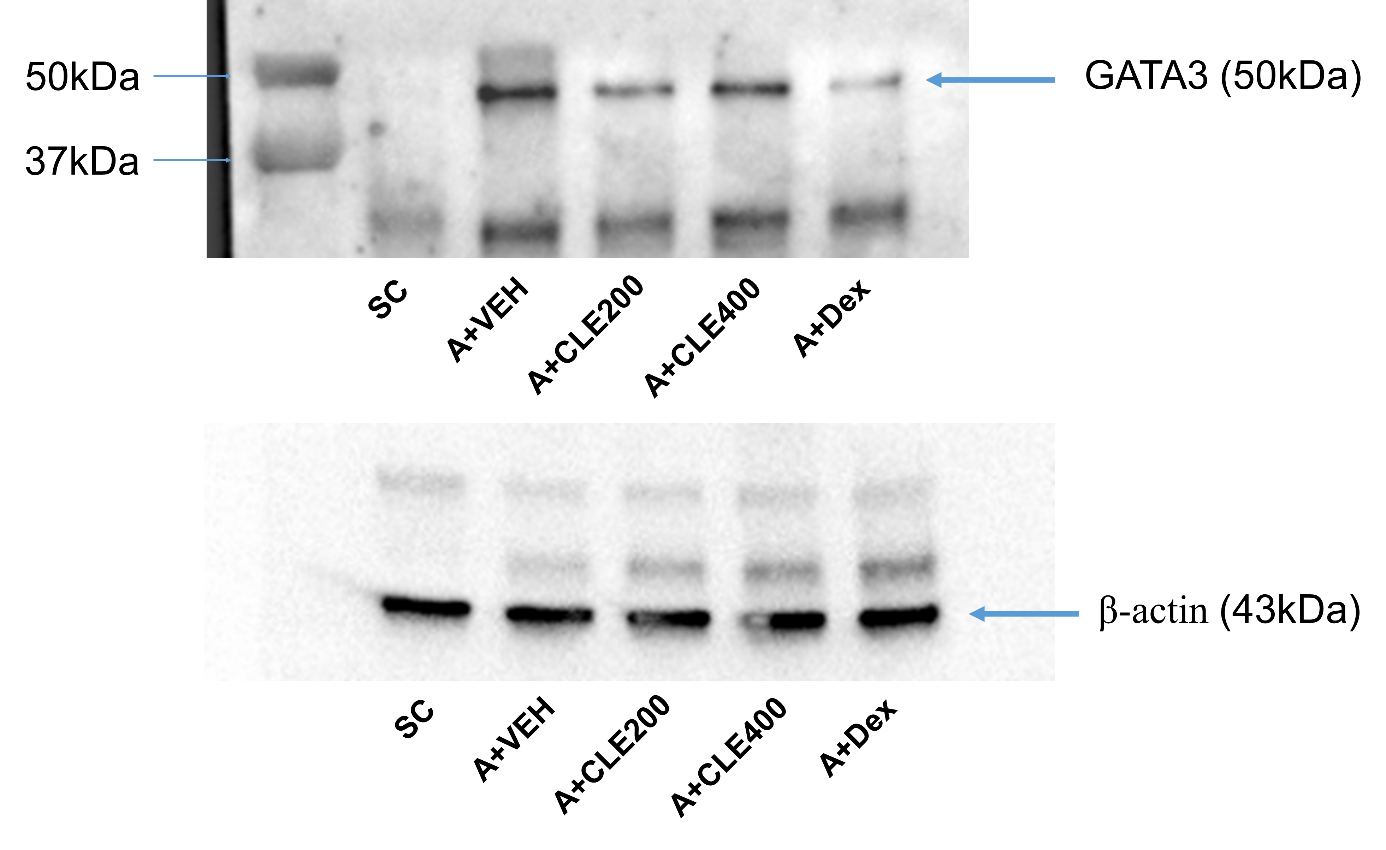
**

**Supplementary Figure S4.**

**
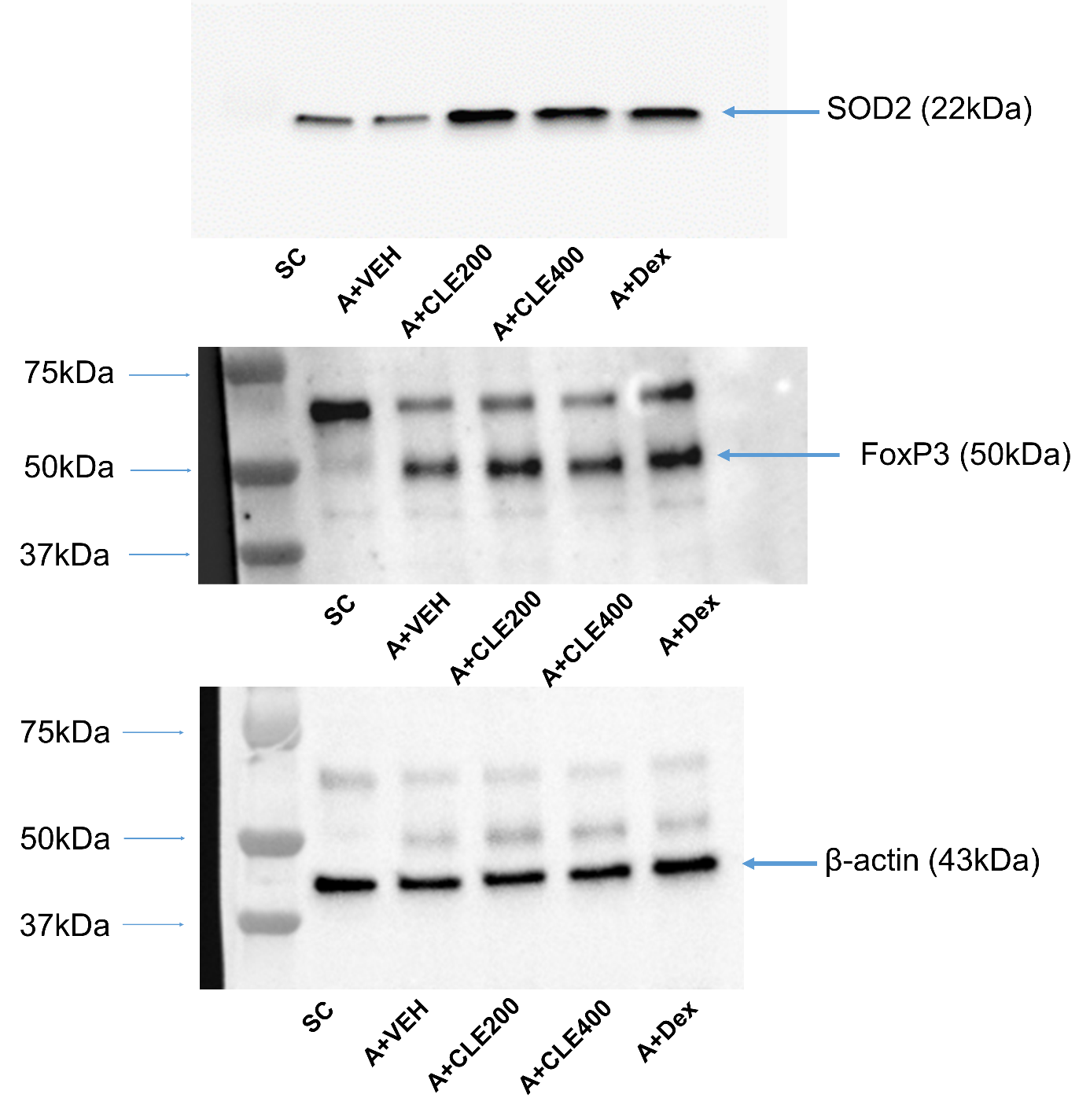
**

**Supplementary Figure S5.**
